# An Extended Chemical Plant Environmental Protection Game on Addressing Uncertainties of Human Adversaries

**DOI:** 10.3390/ijerph15040609

**Published:** 2018-03-27

**Authors:** Zhengqiu Zhu, Bin Chen, Sihang Qiu, Rongxiao Wang, Feiran Chen, Yiping Wang, Xiaogang Qiu

**Affiliations:** 1 College of System Engineering, National University of Defense Technology, 109 Deya Road, Changsha 410073, China; admin@steven-zhu.me (Z.Z.); s.qiu-1@tudelft.nl (S.Q.); wangrongxiao12@gfkd.edu.cn (R.W.); 15507497024@163.com (F.C.); michael.qiu@139.com (X.Q.); 2 Faculty of Electrical Engineering, Web Information Systems, Mathematics and Computer Sciences, Delft University of Technology (TU Delft), Van Mourik Broekmanweg 6, 2628 XE Delft, The Netherlands; 3 The Naval 902 Factory, Shanghai 200083, China; foolwangrain@126.com

**Keywords:** chemical plant environmental protection game, human cognition, bounded rationality, limited observation, learning curves

## Abstract

Chemical production activities in industrial districts pose great threats to the surrounding atmospheric environment and human health. Therefore, developing appropriate and intelligent pollution controlling strategies for the management team to monitor chemical production processes is significantly essential in a chemical industrial district. The literature shows that playing a chemical plant environmental protection (CPEP) game can force the chemical plants to be more compliant with environmental protection authorities and reduce the potential risks of hazardous gas dispersion accidents. However, results of the current literature strictly rely on several perfect assumptions which rarely hold in real-world domains, especially when dealing with human adversaries. To address bounded rationality and limited observability in human cognition, the CPEP game is extended to generate robust schedules of inspection resources for inspection agencies. The present paper is innovative on the following contributions: (i) The CPEP model is extended by taking observation frequency and observation cost of adversaries into account, and thus better reflects the industrial reality; (ii) Uncertainties such as attackers with bounded rationality, attackers with limited observation and incomplete information (i.e., the attacker’s parameters) are integrated into the extended CPEP model; (iii) Learning curve theory is employed to determine the attacker’s observability in the game solver. Results in the case study imply that this work improves the decision-making process for environmental protection authorities in practical fields by bringing more rewards to the inspection agencies and by acquiring more compliance from chemical plants.

## 1. Introduction

In general, air pollutants emitted from factories located in industrial districts are potentially hazardous to surrounding environments and affect human health, materials, as well as forestry [1]. Indeed, long-term violations of air quality standards with respect to industrial production emissions have posed a great threat to the management of a chemical industrial cluster. And in most circumstances, the spontaneous or anthropogenic industrial production emission activities are highly possible to result in atmospheric pollution incidents which can exert harmful or fatal effects on humans and natural environment [2]. Therefore, controlling atmospheric pollution caused by the industrial production is critical to ensure the health of the local residents and the surrounding environment. Due to the unprecedented expansion of chemical industrial activities and the deterioration of atmospheric environment, it is urgent for environmental protection authorities to come up with effective atmospheric pollution controlling measures that can ensure safe production, provide intelligent decisions, and maintain social stability [3,4].

To that end, a series of measures and actions [5,6] have been contributed to abate atmospheric pollution which is caused by the industrial production. Whereas, previous work in addressing this issue falls through generating pragmatic and efficient solutions for environmental protection authorities to optimize audit and detection practices on infraction emissions of chemical plants.

Inspired by several successful game theoretical applications in the security domain [7], Zhu et al. [8] proposed a game theoretic model for optimizing audits and detections of illegal discharge of wastes in chemical clusters, namely, the chemical plant environmental protection (CPEP) game. Following the classical paradigm of Stackelberg security games (SSGs), the CPEP game also assumes perfect rationalities of the game players and complete (and perfect from the attacker’s perspective) information of the game. However, these assumptions rarely hold in real-world domains, especially when dealing with human adversaries. Instead, the decisions of human adversaries may be governed by their bounded rationality [9], which causes them to deviate from their expected optimal strategy. Besides, human adversaries may also suffer from the limited observations of the leader’s mixed strategy (i.e., the game leader’s strategy in the Stackelberg game), giving them a false impression of the leader’s strategy [10]. Thus, a human adversary may not respond with the optimal choice in the game-theoretic concept, causing the leader to face uncertainty over the gamut of attacker’s action. Consequently, when dealing with real uncertainties in human cognition, the CPEP game is supposed to consider two more types of uncertainties: (i) the adversaries’ bounded rationalities; (ii) the adversaries’ limited observation on the defender’s strategies, both of which would affect the adversaries’ best responses.

To address these challenges, realistic models of human behaviors must be integrated into game-theoretic algorithms. Combined observability and rationality assumption (COBRA), introduced by Pita et al. [11], was proved to perform well when interacting with human adversaries. This game solver incorporates the ε-optimal theory [12] and anchoring theory at the meantime. Specifically, ε-optimal theory refers to that the human adversary is boundedly rational, and may not strictly maximize his own utility. As a result, the attacker may select an ε-optimal response [11]. Anchoring theory is referred to as giving full support to the ignorance prior [13], wherein support theory has been adopted to introduce anchoring biases [13,14,15]. Thus, humans would leave some support, α  ∈  [0,  1], on the ignorance prior and the rest, 1−α, on the occurrence they have actually viewed. However, the modeling of how to determine the observation level (i.e., the value of parameter α) still remains an issue.

Some robust game solvers, namely, best response to quantal response (BRQR) [16,17] and robust prospect theory (RPT) [9], were proved to outperform COBRA in most reward structures. However, the applicability of BRQR and RPT to security games and their comparison with COBRA remain open questions [18]. Not only that, one common feature of these human behavioral models is that the parameters in the weighting probability function ought to be learnt from available attacker behavior data. Nevertheless, it is nearly impossible in our study to collect attacker behavior data at this stage. Apart from the methods addressing developing robust game solvers, there was another direction to tackle the problem of human rationality and algorithmic efficiency. Daphne and Brian [19] introduced a new representation language for multi-player games, named *multi*-*agent influence diagrams* (*MAIDS*). It offers a decomposition technique contributing to substantial savings in the computational cost. Based on *MAIDS*, networks of influence diagrams (NID) were presented to describe conflicting and cyclic belief structures, and certain forms of bounded rationality [20]. Combing statistical risk analysis (i.e., influence diagram combined with risk assessment and risk management) with game theory, Insua et al. [21] put forward adversarial risk analysis to deal with analysis of decision-making when there are intelligent opponents and uncertain outcomes. However, these researchers did not provide an ideal paradigm to deal with adversary response uncertainties (i.e., bounded rationality and limited observability) and incomplete information about adversary reward at the same time.

Therefore, the present work follows the research line of COBRA, but is innovative by integrating the learning curve theory [22,23] and by taking the observation costs and observation frequency into consideration. In this way, the extended CPEP game is able to generate a robust daily inspection plan for optimal utilization of inspection resources against human adversaries (e.g., the holders of chemical plants). The robust plan aims to detect infraction of chemical emissions and further reduce incidents’ risks, as well as control air pollution.

The organization of this paper is as follows: Section 2 briefly introduces several baseline works. The main modeling process of the extended CPEP and the corresponding robust game solver are illustrated in Section 2, as well. Case study is realized in Section 3, to illustrate how the extended CPEP and the robust algorithms proposed in this paper perform. Finally, discussions and conclusions are drawn, respectively, in Section 4 and Section 5.

## 2. Model Description

In this section, firstly, baseline models, including Bayesian Stackelberg security games and the CPEP game, are briefly introduced in Section 2.1; then, the extended CPEP game is constructed in Section 2.2; subsequently, Section 2.3 illustrates the modified robust game solver—COBRA, algorithmic complexity, and the combination with learning curve.

### 2.1. Baseline Models

This paper is a follow up of previous research, namely the CPEP game [8]. In this subsection, the general definition of the Bayesian Stackelberg security game and the game-theoretic model—CPEP based on the paradigm of the Bayesian Stackelberg game—are briefly introduced. More details can be found in Zhu et al. [8].

#### 2.1.1. Bayesian Stackelberg Security Games

In a Bayesian game of several agents, each agent *n* must be one of a given set of types. In the present paper, there might be multiple types of attackers (e.g., holders of chemical plants or disgruntled employees), and thus, a Bayesian Stackelberg Game should be employed. In this game, it contains two adversarial agents: the defender and the attacker. Based on real situations, we assume that there is only one defender type (e.g., only one inspection agency), although there are multiple attacker types, denoted by l∈L. However, the defender does not know the attacker’s type. For each agent (defender or attacker) *n*, there is set of strategies $\sigma_{n}$ and a utility function $u_{n}:\;\sigma_{1}\times \sigma_{2}\times L\to ℜ$. The goal is to find the optimal mixed strategy for the defender to gain so-called “First-Mover Advantage” (i.e., though the defender implements her strategy first, she would acquire payoff from a Stackelberg security game no less than that from a simultaneous game) given that the attacker knows this mixed strategy when choosing his own optimal strategy.

#### 2.1.2. The CPEP Game

In a CPEP game, two kinds of adversarial agents are modeled, and they are the inspection agency and the chemical plants. The holders of chemical plants attempt to discharge excessive atmospheric pollutants to acquire more profits after observing the actions taken by the inspection agency (hereafter, we use “leader” or “defender” to refer to the inspection agency, and “follower” or “attacker” to refer to the chemical plant in the remainder of this paper). There are two tasks for the defender: one is to better plan the operating schedules of high-accuracy air quality monitoring stations to obtain more compliance from the chemical plants; and meanwhile, the other task is to reduce its own operational costs of detecting resources. In this paper, the inspection agency and a chemical plant are denoted by notations Θ and Ψ, respectively.

Within the context of a chemical cluster, the pure strategy of both players in the previous paper (i.e., hereafter, the previous paper is referred to Zhu et al. [8]) is modeled as a binary choice in different time slices in one day (i.e., with respect to (w.r.t.) the inspection agency, it is opening the monitoring stations or closing the monitoring stations; for the chemical plants, it is discharging excessive atmospheric pollutants without purification process or not). In this paper, we also assume that one day is equally divided into |T| time slices and the defender has |R| monitoring stations for detection practices (i.e., |R| high-accuracy inspection resources). For the sake of clarity, defender’s (attacker’s) index set of pure strategy can be denoted as $S_{\Theta }$($S_{\Psi }$). Thus, the pure strategy set of both players can be represented by $\sum_{\Theta }=\{\theta_{1},\ldots ,\theta |S_{\Theta }|\}$ and $\sum_{\Psi }=\{\psi_{1},\ldots ,\psi |S_{\Psi }|\}$, respectively. The formulated representations of $\theta_{i}$, $\psi_{j}$, $\left|S_{\Theta }\right|$ and $\left|S_{\Psi }\right|$ are shown in the following formulas:

(1) $$\theta_{i}=\Pi_{r\in R,t\in T}\;s_{d}(r,t),$$

(2) $$\psi_{j}=\Pi_{t\in T}\;s_{a}(t),$$

(3) $$|S_{\Theta }|\;=\;2^{\left|R|\cdot |T\right|},$$

(4) $$|S_{\Psi }|\;=\;2^{\left|T\right|},$$

where $\theta_{i}$ represents a pure strategy for the defender, while $\psi_{j}$ denotes a pure strategy for the attacker; the notations of i and j denote the subscripts of a pure strategy for the defender and the attacker respectively; the notation of R denotes R  =  {1,  2, …, |R|}; similarly, the parameter of T means T = {1,  2, …, |T|}; the notation of $s_{d}(r,t)$ means $s_{d}(r,t)\in \{open,close\}$ while the notation of $s_{a}(t)$ means $s_{a}(t)\;\;\in \;\{release,\;\;no\;release\}$; the cross product is denoted through Π; the number of pure strategies for the inspection agency and the chemical plant are denoted through $\left|S_{\Theta }\right|$ and $\left|S_{\Psi }\right|$, respectively.

For the sake of better illustration, an example of pure strategies in one day for both players is listed in Table 1, if time slice |T| in one day is set at two, and inspection resource |R| is set at one. Further, a mixed strategy is an assignment of a probability to each possible pure strategy. From the point view of a SSG, the defender tends to mix her strategies for more reward, rather than taking a pure strategy. Therefore, the notation $x_{i}$ (i.e., $x_{i}\in [0,1]$) is used to indicate the probability of the defender utilizing the pure strategy $\theta_{i}\in \sum_{\Theta }$. By contrast, the attacker responds after observing the defender’s mixed strategy, and thus, he will take the optimal pure strategy rather than mixing his strategy. To this end, we use $q_{j}\in \{0,1\}$ to indicate the probability of the attacker utilizing the pure strategy $\psi_{j}\in \sum_{\Psi }$.

Under a pure strategy tuple of $\left(\theta_{i},\psi_{j}\right)$ in |T| time slices, payoffs are defined in Formulas (5) and (6) for the defender and the attacker respectively. The parameters of $p_{a1}$, $p_{a2}$, $p_{a3}$, and $p_{a4}$ are used to represent the payoffs for the chemical plant under the pure strategy tuple of (release,open), (release,close), (no release,open), and (no release,close), respectively. Similarly, the parameters $p_{d1}$, $p_{d2}$, $p_{d3}$, and $p_{d4}$ are used to represent the payoffs for the inspection agency under the pure strategy tuples mentioned above. Besides, the notation of $N_{k}$ denotes the number of the $k^{th}$ pure strategy tuples (i.e., (release,open), (release,close), (no release,open), and (no release,close)) under the pure strategy tuple of $\left(\theta_{i},\psi_{j}\right)$ in |T| time slices. For elaborated explanations of these parameters in a chemical industrial terminology, interested readers are referred to Zhu et al. [8].

(5) $$u_{d}^{l}(\theta_{i},\psi_{j})=\sum_{k=1}^{4}N_{k}\cdot p_{dk},$$

(6) $$u_{a}^{l}(\theta_{i},\psi_{j})=\sum_{k=1}^{4}N_{k}\cdot p_{ak},$$

(7) $$\sum_{k=1}^{4}N_{k}=2^{\left|T|\cdot (|R|+1\right)}\;\forall N_{k}\in [0,2^{\left|T|\cdot (|R|+1\right)}]\;and\;N_{k}\in Z,$$

The function of Formulas (5) and (6) is to calculate the summation of each product, that is, the multiplication of the number of the $k^{th}$ pure strategy tuples with the corresponding payoff. Moreover, the utility in the circumstance of mixed strategy for the defender and the attacker can be formulated as follows:

(8) $$u_{d}^{l}(x,q)=\sum_{i\in S_{\Theta }}\sum_{j\in S_{\Psi }}u_{d}^{l}(\theta_{i},\psi_{j})\cdot x_{i}\cdot q_{j}^{l},$$

(9) $$u_{a}^{l}(x,q)=\sum_{i\in S_{\Theta }}\sum_{j\in S_{\Psi }}u_{a}^{l}(\theta_{i},\psi_{j})\cdot x_{i}\cdot q_{j}^{l},$$

In a one-shot game, when the chemical plants are expanded to many types, the payoff for the inspection agency is converted to Formula (10):

(10) $$u_{d}(x,q^{1},\ldots ,q^{\left|L\right|})=\sum_{l}p^{l}\cdot u_{d}^{l}(x,q^{l})=\sum_{l}p^{l}\cdot \sum_{i\in S_{\Theta }}\sum_{j\in S_{\Psi }}u_{d}^{l}(\theta_{i},\psi_{j})\cdot x_{i}\cdot q_{j}^{l},$$

where $q^{l}\;(l\;\;=\;\;1,\;\ldots ,\;|L|)$ defines an assignment of a probability to the $l_{th}$ attacker’s each possible pure strategy (i.e., give a full preference of 100% probability to the optimal pure strategy and leave 0% probability to the rest pure strategies); and $p^{l}$ indicates the prior probability that the $l_{th}$ attacker would occur.

In a CPEP game, a Strong Stackelberg Equilibrium (SSE) solution [24] and a Bayesian Stackelberg Equilibrium (BSE) solution [25] are defined as solution concepts, and interested readers can find the more detailed definition about these two solutions in the previous paper.

### 2.2. Extended CPEP Model

The envisioned extended CPEP game should provide a more realistic approach for the defender (i.e., the inspection agency) to cope with interactions with the human adversaries, that is, the holder of chemical plants or even the disgruntled employees. To achieve this aim, some works should be carried out: (i) In a real industrial scene, chemical plants tend to discharge excessive atmospheric pollutants during each production process, namely, in a game theoretic terminology, the attack is quite frequent against the inspection agency. Thus, the CPEP game should be extended into a more realistic game-theoretic form to model the frequent interactions between defender and attacker; (ii) For one thing, attackers may have limited observation of the inspection agency’s strategy, and thus, he may not perceive the probability distribution over the defender’s mixed strategies correctly. For another thing, the human adversaries, especially the disgruntled employees, may not be utility maximizers (i.e., this kind of attacker is the so-called irrational human adversary). Instead, their preference models may be governed by their bounded rationality [9]. Thus, within the context of a chemical cluster, chemical plants may not respond with a game-theoretic rational strategy, but rather, may have another preference based on bounded rationality or observational uncertainty, and cause the inspection agency to face uncertainty over the gamut of chemical plants’ actions. To deal with this issue, the CPEP game should take attacker’s bounded rationality and limited observability into account.

#### 2.2.1. Players

In the current research, the defender is still the inspection agency, while one more types of attacker (i.e., the disgruntled employees) is considered apart from the holders of chemical plants. Define L as the set of possible attacker types, for instance, L= {different holders of chemical plants; disgruntled employees}. The latter attacker type attempts to discharge atmospheric pollutants on purpose to cause environmental degradation and economic losses. Thus, bounded rationality is always accompanied with this attacker type. Moreover, the defender is assumed rational, while the attackers are assumed bounded rational, based on the following considerations. Firstly, the inspection agency in an extended CPEP is able to perceive her own situation and the opposite player’s actions accurately, while the holder of chemical plants or disgruntled employees may not perceive the opposite player’s actions accurately, due to bounded rationality and limited observation. Secondly, the inspection agency aims to maximize her payoff through the approach of randomly but strategically turn on or off the monitoring stations, but attackers may not be fully utility maximizers. Meanwhile, the interactions between the defender and the attacker in a chemical cluster are characterized by the common knowledge, as explained in Zhu et al. [8]. In this article, the defender and the attacker are also denoted by notations Θ and Ψ, respectively.

#### 2.2.2. Strategies

In the extended CPEP game, the pure strategy of both players is invariant compared to that in a CPEP game; that is, according to Formula (1), the defender’s pure strategy is defined as a combination of operation states of different monitoring stations in all time slices in a day. Similarly, a pure strategy of the attacker is defined as a combination of discharging states in all times slices in a day according to Formula (2). Moreover, it is critical to decide how to make a division of one day because the division method determines how many pure strategies that the defender and the attacker will have. Therefore, our previous research offers a reasonable approach to determine the value of time slices in one day. Interested readers can also employ other reasonable approaches to complete this work.

#### 2.2.3. Payoffs

In this sub-subsection, observation frequency and observation costs are modeled into the extended CPEP game to deal with limited observability of an attacker. To calculate the payoffs of both players from the point view of the environmental protection authority, some parameters explained henceforth are introduced.

As introduced above, there are |L| types of attackers in a chemical cluster. To conduct surveillance on these chemical plants, an inspection agency is equipped with $N_{ms}$ high-accuracy air quality monitoring stations, and a large number of gas sensor modules spread all over the chemical cluster. The successful detection probability of the irregularities of chemical plants through source estimation methods [26,27,28,29], with only the discharging data from the gas sensor modules, is defined as $\gamma_{1}$, while the successful detection probability with the discharging data, from the integrated information of monitoring stations and the gas sensor modules, is defined as $\gamma_{2}$. Clearly, the value of $\gamma_{2}$ is larger than that of $\gamma_{1}$ because the measurements from the high-accuracy air quality monitoring stations are more accurate and reliable.

The definitions in this paragraph are different from those in a CPEP game. In a time slice, the operation costs of high-accuracy air quality monitoring stations are defined as $N_{ms}\cdot C_{d}$, while the operation cost of a purification treatment plant (PTP) for cleansing atmospheric pollutants for a chemical plant is $C_{a}$. As we have investigated, the operational cost of PTPs in a time slice is higher than that of high-accuracy air quality monitoring stations. Before committing to his pure strategy, an attacker is assumed to have a close observation of the defender’s mixed strategy in each round game. Thus, when the game progresses to the *m* round, the total cost of the observation for an attacker is denoted by $(m-1)\cdot C_{o}$. That is, the observation cost in a round is represented by $C_{o}$, and there is no observation experience in the first round of game. Gradually, the attacker would gain more accurate perception of the defender’s mixed strategies after several rounds’ observation.

Moreover, if the $l^{th}$ attacker discharges atmospheric pollutants without purification process, and the inspection agency fails to detect the infraction behavior, then the attacker obtains a reward denoted by $R_{l}^{a}$ from escaping from running PTPs while the inspection agency gets a penalty denoted by $P_{l}^{d}$ from many sources (e.g., environmental impacts, fatalities or injuries, and damage to reputation or negative publicity); conversely, if the inspection agency successfully detects the infraction behavior, then the attacker receives a penalty, $P_{l}^{a}$, while the inspection agency achieves a reward $R_{l}^{d}$. The penalty on an attacker’s infraction behavior mainly comes from forfeits. Part of the fine will be served as a reward for the brilliant work of the inspection agency. Thereby, it is reasonable to assume that $0\leq -P_{l}^{d}\leq R_{l}^{d}$ and $0\leq R_{l}^{a}\leq -P_{l}^{a}$.

To better illustrate, an example of a payoff matrix is listed in Table 2. In this payoff matrix, the attacker is the row player, while the defender is the column player. Thus, we can utilize a parameter $\left(u_{a},u_{d}\right)$ to denote the payoff tuples. The example reveals the binary choice for the defender (e.g., only one inspection resource is considered) and the attacker (i.e., only one type of attacker is considered) in a time slice.

The explanation of this payoff matrix is similar to that in the previous paper. Therefore, we would not explain the duplicated parts in the table again, but focus on the differences. For the defender, in the first case, when the strategy tuple from the attacker and the defender is {release,open}, the payoff is formulated as the reward of a successful detection by high-accuracy air quality monitoring stations and gas sensor modules plus the penalty of unsuccessful detection by the inspection agency minus the operational costs of the high-accuracy air quality monitoring stations through the formula $\gamma_{2}\cdot R_{l}^{d}+(1-\gamma_{2})\cdot P_{l}^{d}-N_{ms}^{}\cdot C_{d}$. In the third case, when the strategy tuple is {no release,open}, the payoff is quite easy, denoted as $-N_{ms}\cdot C_{d}$. The payoffs for the attacker in four circumstances are different from that in the CPEP game because of the application of observation frequency and observation cost. In the first circumstance, we define the payoff for the attacker as $(1-\gamma_{2})\cdot R_{l}^{a}+\gamma_{2}\cdot P_{l}^{a}-(m-1)\cdot C_{o}$. It is computed as the reward of successfully discharging excessive atmospheric pollutants plus the penalty of unsuccessful infraction under the probability $\gamma_{2}$ and minus the observation costs during the previous *m* round game. The difference for the attacker to compute his payoff in the second case is the probability, denoted as $\gamma_{1}$, compared to the first circumstance. The payoffs for the chemical plant are simple in the third and fourth cases, both denoted as the purification cost of $-C_{a}$ plus the observation cost of $-(m-1)\cdot C_{o}$.

Then, Formulas (5)–(7) can also be used to compute the payoffs for both players under a pure strategy tuple of $\left(\theta_{i},\psi_{j}\right)$ in *T* time slices. The only difference is that the parameters of $p_{a1}$, $p_{a2}$, $p_{a3}$, and $p_{a4}$ are changed, because the observation frequency and the observation cost are modeled into the game. Analogously, Formulas (8) and (9) can be considered as payoffs for both players in the circumstance of mixed strategy. Moreover, Formulas (9) and (10) are used to represent the payoffs in a Bayesian Stackelberg extended CPEP game.

Furthermore, in a finite *M* round game, the utility for a defender and several types of attackers can be formulated in the following equations:

(11) $$\begin{array}{l} U_{d}=\sum_{m=1}^{M}u_{d}^{m}(x^{m},q^{m,1},\ldots ,q^{m,|L|})=\sum_{m=1}^{M}\sum_{l}p^{l}\cdot u_{d}^{l}(x^{m},q^{m,l})\\ =\sum_{m=1}^{M}\sum_{l}p^{l}\cdot \sum_{i\in S_{\Theta }}\sum_{j\in S_{\Psi }}u_{d}^{m,l}(\theta_{i},\psi_{j})\cdot x_{i}^{m}\cdot q_{j}^{m,l} \end{array},$$

(12) $$U_{a}^{l}=\sum_{m=1}^{M}u_{a}^{m,l}(x^{m},q^{m,l})=\sum_{m=1}^{M}\sum_{i\in S_{\Theta }}\sum_{j\in S_{\Psi }}u_{a}^{m,l}(\theta_{i},\psi_{j})\cdot x_{i}^{m}\cdot q_{j}^{m,l},$$

where $U_{d}$ indicates the total payoffs during *M* rounds game against |*L|* types of attackers for the defender, while $U_{a}^{l}$ represents the total utility during *M* rounds game against the defender for the $l_{th}$ attacker.

#### 2.2.4. Solution Concepts of the Extended CPEP Game

In a CPEP game, the SSE and BSE are defined as the solution concepts. In contrast, the extended CPEP game is a more realistic game-theoretic model addressing real uncertainties in human cognition, rather than a formal Stackelberg game or a formal Bayesian Stackelberg game. Hence, the solution concepts in the previous research are no longer applicable in the extended CPEP game. 

We draw inspirations from the robust game theory which was first introduced for Nash equilibria [30,31] and further incorporate the learning curve theory with the corresponding solver, COBRA, making predictions about how choices will deviate from the optimal, due to observational uncertainty and bounded rationality. It is noteworthy that the solution concept in this paper is denoted as a robust CPEP equilibrium.

### 2.3. Robust Algorithm

#### 2.3.1. COBRA(α,ε)

In many real scenarios, defenders would face human adversaries who may not take the optimal strategy to respond: this may be caused by their bounded rationality or their limited observability regarding the leader’s strategy. To remedy this situation, Pita et al. [11] drew inspiration from robust optimization methodology [30,32,33] and psychological support theory [34] and introduced a mixed-integer linear program (MILP), COBRA, which builds on the Bayesian Stackelberg game model in decomposed optimal Bayesian Stackelberg solver (DOBSS) [35]. That is, when the two parameters in the COBRA are assigned with values of zero, COBRA is equal to DOBSS. The solver not only deals with reward uncertainty, but also addresses the adversaries’ uncertainty on choosing the expected optimal strategy due to bounded rationality and limited observations. Namely, for one thing, it introduces the idea of robust responses to ε-optimal attacker responses [12] into DOBSS and Stackelberg games in general. For another thing, it also utilizes the concept of anchoring biases [13,14,15] to protect against limited observation conditions, handling observational uncertainty. For the robust algorithm COBRA(α,ε), α and ε represent two parameters that can be adjusted: ε is a parameter related to bounded rationality, while α is a parameter relevant to anchoring bias. Given prior probabilities $p^{l}$ for attackers, the inspection agency solves the following problem formulation:

(13) $$\max_{x,h,q,a,\gamma }\;\;\sum_{l\in L}p^{l}\cdot \gamma_{l},$$

(14) $$s.t.\;\;\;\;\;\sum_{i\in S_{\Theta }}x_{i}=1\;\;,$$

(15) $$\sum_{j\in S_{\Psi }}h_{j}^{l}\geq 1\;\;\forall l\in L,$$

(16) $$\sum_{j\in S_{\Psi }}q_{j}^{l}=1\;\;\forall l\in L,$$

(17) $$0\leq (a^{l}-\sum_{i\in S_{\Theta }}C_{ij}^{l}\cdot x_{i}^{\prime })\leq (1-q_{j}^{l})\cdot M_{R}\;\;\forall l\in L,j\in S_{\Psi },$$

(18) $$\varepsilon \cdot (1-h_{j}^{l})\leq a^{l}-\sum_{i\in S_{\Theta }}C_{ij}^{l}\cdot x_{i}^{\prime }\leq \varepsilon +(1-h_{j}^{l})\cdot M_{R}\;\;\forall l\in L,j\in S_{\Psi },$$

(19) $$M_{R}\cdot (1-h_{j}^{l})+\sum_{i\in S_{\Theta }}R_{ij}^{l}\cdot x_{i}\geq \gamma_{l}\;\;\forall l\in L,j\in S_{\Psi },$$

(20) $$q_{j}^{l}\leq h_{j}^{l}\;\;\forall l\in L,j\in S_{\Psi },$$

(21) $$h_{j}^{l},q_{j}^{l}\in \{0,1\}\;\;\forall l\in L,j\in S_{\Psi },$$

(22) $$x_{i}\in [0,1]\;\;\forall i\in S_{\Theta },$$

(23) $$a^{l}\in ℜ\;\;\forall l\in L,$$

(24) $$x_{i}^{\prime }=\alpha \cdot (1/|S_{\Theta }|)+(1-\alpha )\cdot x_{i}\;\;\forall i\in S_{\Theta },$$

where $\gamma_{l}$ is set to the minimum defender reward allowing COBRA(α,ε) to robustly cope with the worst scenario; the perceived defender’s strategy by the attacker is denoted by $x_{i}^{\prime }$, where $x_{i}^{\prime }$ is defined by the linear model presented in the Formula (24), which is a combination of the support for real defender’s strategy $x_{i}$ and the support toward the ignorance prior. Thus, given this knowledge, the defender can find the attacker’s responses based on $x_{i}^{\prime }$ and optimize its actual strategy $x_{i}$ against this strategy; the value $x_{i}$ is the proportion of times in which pure strategy $\theta_{i}\in S_{\Theta }$ is used in the policy. Similarly, we denote, by $q_{j}^{l}$, the optimal strategy for attacker type l∈L over the possible pure strategies with a maximum reward of $a^{l}$ in the Formulas (16) and (17). Variables $h_{j}^{l}$ represent all ε-optimal strategies for attacker type l; the second constraint (i.e., Formula (15)) allows selection of more than one ε-optimal strategy per attacker type. Let $M_{R}$ be a large positive number (i.e., the large number along with the definition of $q_{j}^{l}$ and $h_{j}^{l}$ ensures that $q_{j}^{l}=1$ only for a strategy *j* that is optimal for attacker type *l*. By this way, it works as a branch-and-bound method to find solutions in MILPs and narrows the solution space greatly. The large number is usually set between 10^4^ and 10^6^. We also index the payoff matrices of the defender and each of the attacker types l∈L by the matrices $R^{l}$ and $C^{l}$, where $R_{ij}^{l}$ and $C_{ij}^{l}$ are rewards obtained if the leader takes strategy $\theta_{i}\in S_{\Theta }$ and the attacker type l takes the strategy $\psi_{j}\in S_{\Psi }$. The fifth constraint (i.e., Formula (18)) ensures that when $h_{j}^{l}=1$ meets for every action *j* such that $a^{l}-\sum_{i\in S_{\Theta }}C_{ij}^{l}\cdot x_{i}^{\prime }\leq \varepsilon$, since in this case, the middle term in the inequality is less than ε, and the left inequality is then only satisfied if $h_{j}^{l}=1$. The sixth constraint (i.e., Formula (19)) helps define the objective value against attacker type l, $\gamma_{l}$, which must be lower than any defender’s reward for all actions $h_{j}^{l}=1$, as opposed to the DOBSS formulation, which has only one action $h_{j}^{l}=1$.

#### 2.3.2. Complexity

The problem of finding an optimal solution in a Bayesian Stackelberg game has been demonstrated to be a NP-hard task in [25]. Thus, the solver, COBRA(α,ε) in this paper, along with the previous used solver, DOBSS, in the previous paper, is a MILP that faces a NP-hard problem. A number of well known, efficient solution packages or software (e.g., CPLEX, YALMIP, MATLAB) for MILPs can be used to solve this problem, and their calculation performances rely on the number of integer variables. Specifically, DOBSS considers |Q||L| integer variables (i.e., a multiplication of quantity of pure strategy from attackers and quantity of attacker types) while COBRA(α,ε) doubles that. In the experimental section, another robust solver named MAXIMIN [11] is also used to compare solution performances. MAXIMIN is a linear programming problem which can be solved in a polynomial time. Thus, it is obvious that MAXIMIN will have the lowest running time per problem instance, while COBRA(α,ε) takes the longest running time. In this paper, all experiments are run on a machine with an Intel Core 2.6 GHz processor and 8 GB of RAM through MATLAB. So as shown in runtime results, we can observe some of the regularities mentioned above.

#### 2.3.3. Learning Curve

Learnt from the theory of anchoring bias, α can be assigned to a value based on how much evidence that we think the human adversary will have. Namely, if the human attacker is expected to observe our policy frequently and carefully, then α will be low, while if we suspect he will not have many observations of our policy, α will be high. However, the COBRA is an MILP-based algorithm, and the optimal solutions will situate on the boundary of feasible space. Therefore, an incorrect value of α may lead to a severe degradation to the defender’s payoff. Previous research in this domain did not cover the design of a reasonable model for the parameter α. As is known to all, learning is the process of acquiring new or modifying existing knowledge, behaviors, skills or preferences. Evidence shows that some learning is immediate, induced by a single event (e.g., being burned by a hot stove), but much knowledge, as well as skill, accumulates from repeated experience [36]. Among different kinds of learning types, observational learning is learning that occurs through observing the behavior of others [37]. It is a form of social learning which takes various forms, based on a constant process [38]. Therefore, the judgment of attackers on the defender’s strategy can be considered as an observational learning process.

**Figure 1:**
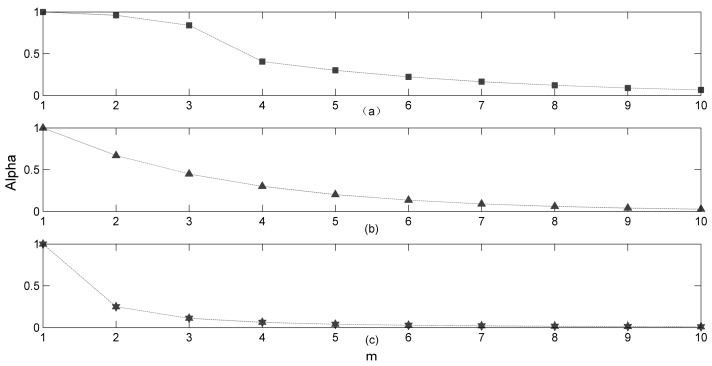
Learning curves under different functions (the values of parameters in (**a**) are: *a* = 0.04, *b* = 0, $C_{o}$ = 0.3, $m_{0}$ = 3; the value of parameter in (**b**) is: $C_{o}$ = 0.4; the values of parameters in (**c**) are: $C_{o}$ = 0.2, k = 10).

Learning curve, a term describing that a body of knowledge is learnt over time, is an increase of learning (vertical axis, is often a measure representing learning or proficiency or other proxy for “efficiency” or “productivity”) with experience (horizontal axis, it often represents experience either directly as time, or can be related to time, like a number of trials). The first known utilization of the term, learning curve, is in 1897: Bryan and Harter found, in their study of the acquisition of the telegraphic language, a learning curve which rose rapidly at the beginning followed by a period of retardation, and was thus convex to the vertical axis [39]. Moreover, Arthur Bills gave a more detailed description of learning curves in 1934 [40]. He also discussed the properties of different types of learning curves, such as negative acceleration, positive acceleration, plateaus, and ogive curves. Several main functions have been used to describe the learning curves in [41,42], and they are S-curve or sigmoid function, exponential growth (i.e., the proficiency can increase without limit), exponential rise or fall to a limit, and power law. Specifically, considering the value range of the parameter α and its variation characteristic, the piecewise functions, the exponential fall functions, and the power law functions are ideal options to model the parameter α. Further, observation frequency and observation cost are modeled in the learning curve function in this paper. The detailed functions are formulated as follows:

(i) The piecewise function:
  (25) $$\alpha =f(m)\;\;=\;\;\left\{ \begin{array}{l} 1-\;(a\cdot {\left(m\;-\;1\right)}^{2}+b\cdot (m-1))\;\;0\leq m\leq m_{0};\;\;a,b\in R^{+}\\ e^{-(m-1)\cdot C_{o}}\;\mathrm{or}\;C_{o}\cdot {\left(m\;-\;1\right)}^{-k}\;\;\;m>m_{0};\;\;m\in Z^{+};\;\;C_{o}\in R^{+} \end{array}\right..$$
  The piecewise function is a combination of different functions, namely, a polynomial function and an exponential fall function or a power law. The parameters a, b, k in the function are constant arithmetic numbers. Moreover, the number of observations is denoted as *m*, while the observation cost is represented by $C_{o}$.
(ii) The exponential function:
  (26) $$\alpha =f(m)=e^{-(m-1)\cdot C_{o}}\;\;m\in Z^{+};\;\;C_{o}\in R^{+},$$
  where the observation frequency *m* is a positive number and the observation cost $C_{o}$ is an arithmetic number.
(iii) The power law function:
  (27) $$\alpha =f(m)=m^{-C_{o}\cdot k}\;\;\;m\in Z^{+};\;\;C_{o}\in R^{+};\;\;k\in R^{+},$$
  For the sake of better illustration, an exemplified figure (Figure 1) of these learning curves for parameter α is shown as below.

## 3. Case Study

In this section, a practical case study implemented in Shanghai chemical cluster is utilized to elaborate and explain how the extended CPEP game works in a real industrial scene to help supervise the chemical production process for the inspection agency.

### 3.1. Basic Settings

**Figure 2:**
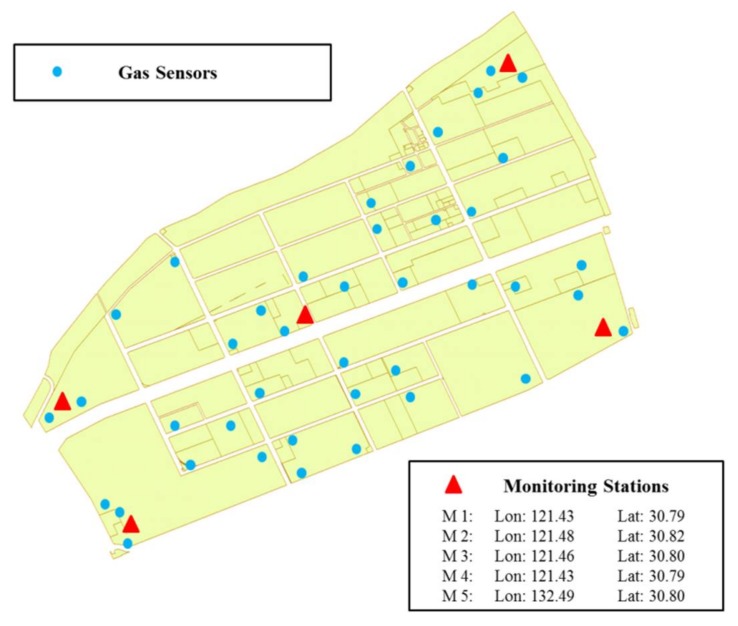
Layout of the case study (the triangles indicate the high-accuracy air quality monitoring stations, while the circles represent gas sensor modules in this figure).

Figure 2 shows a typical refinery map of a chemical industrial park in Shanghai, China, which is also the study area used in previous research [8,26]. The detailed information and explanations of this case are also provided in these research studies, and interested readers are referred to these references. Apart from the previously mentioned 23 types of attackers in previous research [8], a new type of human adversary, the disgruntled employees, is considered in this paper as well. Namely, there are a total of 24 types of attackers against the inspection agency. To meet the practical requirements in our modeling process, monitoring stations are open at the same time to collect data, or shut down together to reduce costs, because monitoring data utilized in source estimation methods are required to be diverse, rather than data from only one monitoring station or two monitoring stations. Therefore, five monitoring stations are treated as one resource of inspection agency. More numerical settings of this case study, since most are referred from Zhu et al. [8], are given in Supplementary Materials (Tables S1 and S2).

Pure strategies for different attacker types are the same as those in Table 2. However, the payoffs for these attackers are changing with the corresponding types. Hence, it is difficult to determine parameters of each attacker exactly one by one, owing to the large number of attacker types. To simplify this problem, the experts from the inspection agency help to determine the lower bound and upper bound of some parameters (e.g., penalty for the defender and reward for the attacker). The exact values of these parameters in this paper are set the same as those in the previous research, and related explanations are attached. Here, it is worth noting that the information concerns estimations from the defender’s point of view. In the future, utilizing a more realistic way to determine the attacker’s payoff will be another research direction. Then, a series of parameters along with corresponding values are given in Table 3. Besides, it is noteworthy that the prior probabilities with threats of different attacker types are also concluded in Table S2.

In this paper, bounded rationality and limited observability of human adversaries are modeled in the extended CPEP game. Therefore, we aim to explore how bounded rationality or limited observability would impact the decisions and corresponding payoffs for both players. The control variate method is adopted to implement the practical experiments: (i) when the value of epsilon ε denoting the bounded rationality is invariant, the first experiment is conducted to test how different learning curve functions and observation frequency will influence the decision-making process of both players; (ii) when the value of limited observability denoted by α is invariant, the second experiment is conducted to test how biased perception of adversary will impact the decisions of the defender. With the parameters and models above, the extended CPEP game can be solved through the game solver COBRA. Meanwhile, robust solutions computed by the MAXIMIN and previous industrial practice (i.e., in the previous industrial practice, the inspection agency keeps the inspection resources on all the time regardless of the costs, but none of the compliance is acquired without utilizing source estimation methods) are also used to make comparisons with robust CPEP equilibriums.

### 3.2. Results on Different Observational Conditions

In the case that the value of epsilon ε denoting the bounded rationality is set unchanged at 2.5, we examine the impact of three different learning curve functions under different observation numbers on the final decisions and payoffs for both defenders and attackers. Here, a finite M=6 game is considered. Meanwhile, we utilize the piecewise function, exponential fall function, and power law function proposed in Section 2.3.3 (i.e., the values of parameters in the following formulas are from experts of inspection agency) to model the parameter α in sequence and they are formulated as follows:

(28) $$\alpha =f(m)=\left\{ \begin{array}{l} 1-0.03\cdot {\left(m-1\right)}^{2}\;\;1\leq m\leq 3\\ e^{-2\cdot (m-1)}\;\;4\leq m\leq 6 \end{array}\right.,$$

(29) $$\alpha =f(m)=e^{-2\cdot (m-1)}\;\;1\leq m\leq 6,$$

(30) $$\alpha =f(m)=m^{-2}\;\;1\leq m\leq 6,$$

The robust CPEP equilibriums under different observational conditions shown in Table 4 are computed through the COBRA, and corresponding maximum payoffs (i.e., this is the per-period payoff for the defender, but not the total payoffs) and compliance numbers from the attackers for the inspection agency are also listed. In Table 4, the notation of Def Strategy denotes the mixed strategy of the defender, while the notation of Def Payoff means utility of the inspection agency under corresponding mixed strategy. Besides, the notation of Compliance Number indicates the number of chemical plants being compliant with the inspection agency. More elaborations about the attacker’s strategy are concluded in Supplementary Materials (Tables S3–S5).

**Figure 3:**
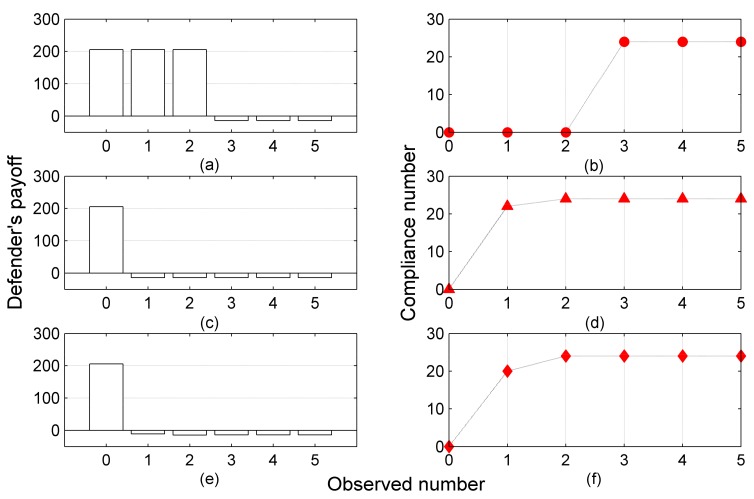
Defender’s payoff and compliance number under different observation number ((**a**) is the defender’s payoff under piecewise function; (**b**) is the compliance number under piecewise function; (**c**) is the defender’s payoff under exponential fall function; (**d**) is the compliance number under exponential fall function; (**e**) is the defender’s payoff under power law function; (**f**) is the compliance number under power law function).

For the sake of better exemplification, we take a row to explain in detail. When the exponential fall function is selected to model the limited observation, and the observation frequency *m* is set at 4 as well, the result in this row indicates that the inspection agency plays the strategy (open,open) at a probability of 0.386, plays the strategy (open,close) at a probability of 0.307, and so forth. In this robust solution, the inspection agency would, on the one hand, obtain a reward of −13.8596 in the fourth day, and on the other hand, acquire compliance from all the attackers. Namely, all the chemical plants are compliant with the inspection agency by choosing the pure strategy of {no release,no release}. The detailed attacker’s payoff and the attacker’s strategy w.r.t. different observation conditions are exhibited in Supplementary Materials (Tables S3–S5). The notation Att_P and Att_Str in Tables S3–S5 represent the attacker’s payoff and the attacker’s strategy, respectively. To draw regularities from this experiment, the Figure 3 w.r.t. results in Table 4 is presented. From the figure, it is not hard to conclude that when the observation number is set at 0 at the first day (i.e., m=1), the attackers are still accustomed to release excessive atmospheric pollutants to the surrounding environment, resulting in enormous profits for the inspection agency, because in this case, the inspection agency chooses to play the pure strategy (open,open). Moreover, with the increase of the observation number, the attackers would learn more information about the defender’s mixed strategy, and thus, they will choose to obey the regulation and process the atmospheric discharges through the PTPs. In these circumstances, the inspection agency would receive lower payoff but more compliance. Meanwhile, robust solutions under different observation conditions computed by the solver, MAXIMIN, are provided in Table 5. It is an obvious conclusion that under the same payoff matrix, the maximum payoff computed by MAXIMIN for the inspection agency is less than that acquired from COBRA. Furthermore, the result proves that the COBRA solver outperforms the MAXIMIN solver in dealing with real uncertainties in human cognition. In the past inspection practice, as defined above, the inspection agency acquired a payoff at −820 RMB, and no compliance from the chemical plants in a one-day game. By comparison, it is obvious that the inspection agency improves her payoffs and acquires more compliance from different types of attackers, along with human uncertainties when the extended CPEP game is played.

Apart from the analysis on payoffs and optimal strategies of both players, runtime results are also listed in Table 4 and Table 5. To analyze our runtime results, some information must be explained at first. The value of large positive number $M_{R}$ in COBRA solver, serving as a branch-and-bound method, determines the size of solution search space. At first, we would set a larger value of $M_{R}$ to narrow the search space and reduce runtime if the optimal solution computed can pass the solution test finally. Otherwise, a smaller value of $M_{R}$ will be chosen to enlarge the search space until the optimal solution can pass the solution test. After finding a reasonable value of $M_{R}$ for each circumstance, we implemented five to ten trials for each circumstance, and the averaged running time is served as runtime result. As expected, MAXIMIN is the fastest among the algorithms with a maximal runtime of 0.0242 s, on average, in our case. However, not anticipated is that some of the runtime results computed by COBRA witness a significant speedup compared with the runtime results calculated by DOBSS in the previous paper. The surprising finding is also concluded in [11]. In that paper, elaborated experiments on runtime among the COBRA, DOBSS, and MAXIMIN were carried out. Interested readers can be referred to it for detailed information. Another interesting but intuitive result in runtime is that when the value of alpha α is large (i.e., the attacker knows little information about the defender’s action), the solution space is small, and the problem can be solved in a very short time. As in this situation, the defender will choose to open the inspection resources without hesitation.

Therefore, playing an extended CPEP game is really essential for the inspection agency in her daily management work, because the game not only reduces her daily operational costs and controls the discharging behaviors of chemical plants, but also extends the inspection practice into a more realistic industrial trial.

### 3.3. Results on Different Rational Conditions

In the case that the value of alpha α denoting the limited observation is set unchanged at 0.5, we examine the influence of different rationality levels of attackers on the final decisions and payoffs for both players. Although the value of epsilon ε is often set larger than 2.5 [11], we are curious about the outcomes in the case where the value of epsilon is set between 0 and 2.5. Therefore, a variety of values for the parameter ε are tested through solving the corresponding COBRA solver. At the meantime, the robust CPEP equilibriums under different rationality levels of attackers are shown in Table 6, and corresponding maximum payoffs and compliance numbers from the attackers for the inspection agency are also listed. The notations in this table are defined the same as those in Table 4. For the sake of simplicity, we will not explain them again here. More elaborated results are concluded in Supplementary Materials (Table S6). The notation Att_P and Att_Str in Table S6 also represent the attacker’s payoff and the attacker’s strategy, respectively. Firstly, it can be derived from Table 6 that when the value of epsilon ε increases, the reward for the inspection agency decreases accordingly. Moreover, the proposition in [11] has proved this finding, and it is organized as the optimal reward of COBRA is decreasing in epsilon ε when observation condition is invariant. Proof of this finding is that since the fifth constraint in Formula (18) makes $q_{j}^{l}=1$ when that action has an attacker reward between $\left(a^{l}-\varepsilon ,a^{l}\right]$, increasing ε would increase the number of attacker strategy set to 1. Having more active attacker actions in the sixth constraint (i.e., Formula (22)) can only decrease the minimum value $\gamma^{l}$.

**Figure 4:**
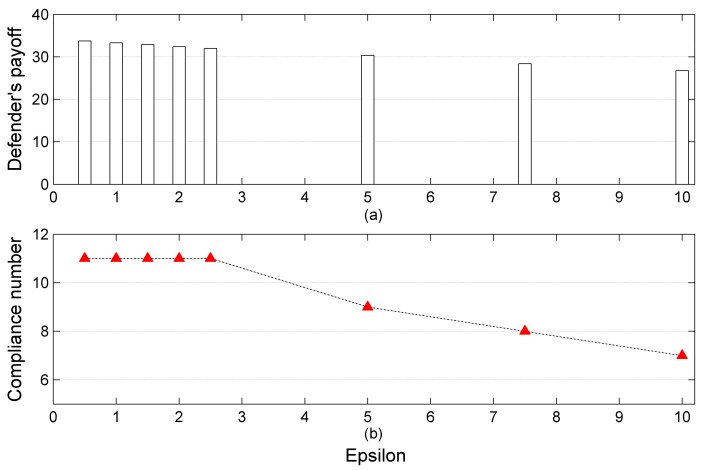
Defender’s payoff and compliance number under different rationality levels of attackers ((**a**) denotes the defender’s payoff when the value of epsilon increases while (**b**) denotes the compliance number in the same case).

Significantly, it is worthwhile to note that when the value of epsilon ε is set between 0 and 2.5, the compliance number from the attackers is unchanged; and the phenomenon indicates that the rational level of attackers in this case can be categorized into the same class. Besides, a critical value of epsilon ε may exist in the interval between 2.5 and 5, leading to the variation in compliance number from the attackers. Moreover, as the value of epsilon ε increases further, the compliance number from the attackers will reduce correspondingly. The phenomenon is consistent with a common sense where attackers with a high level of bounded rationality are more likely to deviate from their optimal choice, rather than comply with the inspection agency. To better illustrate this, the above findings are clearly drawn in Figure 4. The defender’s payoff and compliance number from the attackers are decreasing with the increase of epsilon ε.

**Figure 5:**
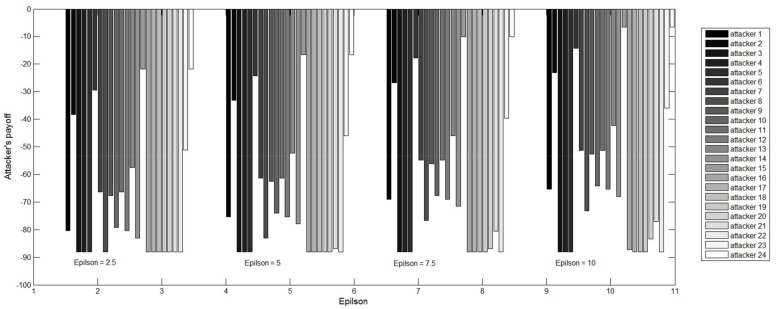
Attacker’s payoff under different rationality levels of attackers.

In addition, the corresponding payoffs of attackers under different rational levels are listed in Table S6 and are drawn in Figure 5. In Figure 5, the figure legend represents different colors with respect to corresponding attacker’s payoff in a combined column. It is concluded that the attacker’s payoff also reduces with the increase of epsilon ε, because the attackers are more likely to discharge excessive air pollutants when the inspection resources are in the working states. Therefore, chemical plants are more likely to be fined for their irregularities.

Meanwhile, the robust solution calculated by MAXIMIN in this experimental setup is also provided. The optimal payoff for the inspection agency is −14.988 at the mixed strategy of [0.562,  0.185,  0.185,  0.068]. It can be seen that the robust CPEP equilibrium is more promising compared with that in MAXIMIN.

Regularities of runtime results found in this experiment are similar to those in the former subsection. Though there are some slight fluctuations, another general trend is that with the increase of the value in epsilon ε, the runtime of optimal solution witnesses a significant rise. Overall, the runtime of a robust CPEP equilibrium computed by COBRA is acceptable compared with that calculated by DOBSS in the previous paper.

Finally, these results reveal that if the attackers are more irrational, the relevant outcomes for both players are not satisfying. Namely, the attackers would receive less reward, while the inspection agency will obtain less compliance from the attackers. Irrational attackers are often less concerned of their payoffs, thereby exposing a great threat to the management work of inspection agency.

## 4. Discussion

Several findings and drawbacks can be concluded from the results of the case study, as shown in the following text.

The first finding is that no matter what kind of learning curve the attacker belongs to, the inspection agency is feasible to achieve compliance from the human adversaries and acquire better payoff than before through playing the extended CPEPs. Learning from the case study, compared to previous industrial practice and robust solution computed by MAXIMIN, robust CPEP equilibriums computed by COBRA are proved to acquire more compliance and higher payoff for the inspection agency. Meanwhile, runtime results are also acceptable compared with those in DOBSS solver. Moreover, more compliance from the attackers indicates that less atmospheric pollution would be discharged, and the surrounding residential environment will be greatly improved.

The second finding is that with the increase of the observation number on the mixed strategy of defender, the inspection agency would acquire more compliance from the attackers. Meanwhile, it is noteworthy that the optimal payoffs and the corresponding equilibriums for both players are different under different learning curve functions. In some learning curves, the attackers will learn the defender’s strategy in a few rounds’ observation, while the situation is just the opposite in other learning curves. Whatever the learning curve is, the robust CPEP equilibriums would bring more benefits, namely, more reward and more compliance, to the inspection agency.

Another finding is that when the value of epsilon denoting the bounded rationality increases, the corresponding payoffs for both players and the compliance from the attackers would decrease at the same time. It reveals that more irrationality in human adversaries only leads to destruction to both sides of players. However, some types of attackers are careless of their payoffs, and thus, it is better to overestimate the dangerous attackers. Even in this extreme circumstance, the extended CPEP game can still ensure higher payoffs and more compliance numbers from chemical plants for the inspection agency.

However, there are also some limitations in our results. The parameters related to the chemical plants are given by domain experts from the inspection agency, the exact value of which may be inaccurate in practical cases. In this circumstance, a game with the defender’s uncertainty on the attacker’s parameters should be played, see for instance, Zhang et al. [43]. Besides, a variety of learning curve functions are used in this study to predict the value of limited observability. However, some practical experiments remain to be carried out to determine the authentic learning curve function for the human adversaries in a chemical industrial domain.

## 5. Conclusions

Adversaries in any setting, also in the chemical industry, contain uncertainties, especially human subjects, and hence, it is reasonable to take the adversary response uncertainties (i.e., bounded rationality and limited observability) and incomplete information about adversary reward (i.e., different reward matrices with a Bayesian a priori distribution assumption) into account. Unfortunately, the lack of data w.r.t. adversaries’ infraction behavior brings huge difficulties for the management layer in a chemical industrial park. Existing game-theoretical models, including the CPEP game for atmospheric environment protection within a chemical cluster, stay at the primary state, assuming perfect rationality and unbiased actions for the attackers. To this end, the extended CPEP game proposed in this article moves forward the state of the art of this domain with the following originalities:

(i) The first originality is reshaping the CPEP game into a more realistic game-theoretic model form, and considering the effect of observation frequency and observation cost on reward matrices;
(ii) The second originality is building uncertainties, such as bounded rationality, limited observation and reward uncertainty (i.e., different adversary’s types) into the extended CPEP game;
(iii) The third originality is incorporating the learning curve with the robust game solver, COBRA, which offers a reasonable method to determine the value of alpha in COBRA.

Results of the case study show that the inspection agency will be able to achieve more compliance from the attackers and improve her payoff through playing the extended CPEPs, compared to the previous industrial practice. The extended CPEP game successfully captured the intelligent interactions between the defender and the adversaries with human cognition uncertainties. Moreover, under the settings of the case study, our robust CPEP equilibrium is proved to outperform the solutions computed by the DOBSS, the MAXIMIN and the previous industrial practice. Furthermore, the defender’s payoff is robust w.r.t. different types of attackers (e.g., irrational attackers or attackers with limited observability) and it is better for the defender to overestimate the dangerous adversaries than to underestimate them. What is pleasantly surprising is that the runtime results computed by COBRA are acceptable, and outperform those computed in DOBSS in some reward structures. Finally, playing the extended CPEP game is bound to improve the surrounding ecosystem and residential environment on the one hand, and to reduce the risks of hazardous gas leakage incidents or accidents considerably on the other hand.

The proposed model can be further developed to consider unknown opponents (i.e., exact values of parameters related to adversaries are unknown), as well as to study the results of source estimation methods to model infraction behaviors of attackers.

## Figures and Tables

**Table 1:** Pure strategy of defender and attacker in one day with two time slices.

Notation	Defender’s Strategy	Notation	Attacker’s Strategy
$\theta_{1}$	{open,open}	$\psi_{1}$	{release,release}
$\theta_{2}$	{open,close}	$\psi_{2}$	{release,no release}
$\theta_{3}$	{close,open}	$\psi_{3}$	{no release,release}
$\theta_{4}$	{close,close}	$\psi_{4}$	{no release,no release}

**Table 2:** Payoff matrix in a time slice with only one defender and one attacker.

	Defender		Close
Attacker			
Release		$(1-\gamma_{2})\cdot R_{l}^{a}+\gamma_{2}\cdot P_{l}^{a}-(m-1)\cdot C_{o}$, $\gamma_{2}\cdot R_{l}^{d}+(1-\gamma_{2})\cdot P_{l}^{d}-N_{ms}\cdot C_{d}$	$(1-\gamma_{1})\cdot R_{l}^{a}+\gamma_{1}\cdot P_{l}^{a}-(m-1)\cdot C_{o}$, $\gamma_{1}\cdot R_{l}^{d}+(1-\gamma_{1})\cdot P_{l}^{d}$
No release		$-C_{a}-(m-1)\cdot C_{o}$,$-N_{ms}\cdot C_{d}$	$-C_{a}-(m-1)\cdot C_{o}$,0

**Table 3:** Value of parameters.

Parameters	Value	Parameters	Value
$C_{d}$	2	$R_{l}^{a}\;\max$	900
$C_{a}$	40	$R_{l}^{a}\;\min$	800
$R_{l}^{d}$	600	$P_{l}^{a}$	−1600
$P_{l}^{d}\;\max$	−350	|L|	24
$P_{l}^{d}\;\min$	−400	$\gamma_{2}$	0.5
$\gamma_{1}$	0.1	|T|	2
|R|	1	$C_{o}$	2
$N_{ms}$	5	M	6

**Table 4:** Results of the extended chemical plant environmental protection (CPEP) game computed by COBRA under different observation conditions (the notation of time represents the running time of the solver and the notation of s in this table denotes seconds).

Learning Curve	Observation Number	Alpha	Def Strategy	Compliance Number	Def Payoff	Time
Piecewise function	*m* = 1	1	(1, 0, 0, 0)	0	205.9485	0.155 s
	*m* = 2	0.97	(1, 0, 0, 0)	0	205.9485	0.099 s
	*m* = 3	0.88	(1, 0, 0, 0)	0	205.9485	0.130 s
	*m* = 4	0.0025	(0.386, 0.307, 0.307, 0)	24	−13.8596	1073.5 s
	*m* = 5	3.3546 × 10^−4^	(0.6926, 0, 0, 0.3074)	24	−13.8474	1460.7 s
	*m* = 6	4.5400 × 10^5^	(0.6925, 0, 0, 0.3075)	24	−13.8474	1568.3 s
Exponential fall function	*m* = 1	1	(1, 0, 0, 0)	0	205.9485	0.183 s
	*m* = 2	0.1353	(0.434, 0.283, 0.283, 0)	22	−14.356	3097.9 s
	*m* = 3	0.0183	(0.392, 0.304, 0.304, 0)	24	−13.9218	3604.8 s
	*m* = 4	0.0025	(0.386, 0.307, 0.307, 0)	24	−13.8596	1085.5 s
	*m* = 5	3.3546 × 10^−4^	(0.6926, 0, 0, 0.3074)	24	−13.8474	1473.6 s
	*m* = 6	4.5400 × 10^−5^	(0.6925, 0, 0, 0.3075)	24	−13.8474	1580.7 s
Power law function	*m* = 1	1	(1, 0, 0, 0)	0	205.9485	0.263 s
	*m* = 2	0.25	(0.478, 0.261,0.261, 0)	20	−10.629	822.56 s
	*m* = 3	0.1111	(0.433, 0.2835, 0.2835,0)	24	−14.3312	2390.9 s
	*m* = 4	0.0625	(0.41, 0.295, 0.295, 0)	24	−14.1067	4372.9 s
	*m* = 5	0.0400	(0.7005, 0, 0, 0.2995)	24	−14.0104	1867.3 s
	*m* = 6	0.0278	(0.698, 0, 0, 0.302)	24	−13.96	3908.3 s

**Table 5:** Results computed by MAXIMIN under different observation conditions (the notation of time represents the running time of the solver and the notation of s in this table denotes seconds).

Observation Number	Def Strategy	Def Payoff	Time
*m* = 1	(0.562, 0.185, 0.185, 0.068)	−14.98797595	0.0223 s
*m* = 2	(0.562, 0.185, 0.185, 0.068)	−14.98797595	0.0242 s
*m* = 3	(0.562, 0.185, 0.185, 0.068)	−14.98797595	0.0100 s
*m* = 4	(0.561975, 0.185019, 0.185019, 0.067987)	−14.988	0.0100 s
*m* = 5	(0.561975, 0.185019, 0.185019, 0.067987)	−14.988	0.0089 s
*m* = 6	(0.561975, 0.185019, 0.185019, 0.067987)	−14.988	0.0099 s

**Table 6:** Results computed by COBRA under different rationality levels of attackers (the notation of time represents the running time of the solver and the notation of s in this table denotes seconds).

Value of Epsilon	Def Strategy	Compliance Number	Def Payoff	Time
ε = 0.5	(0.6358, 0.1821, 0.1821, 0)	11	33.7456	466.3 s
ε = 1	(0.6338, 0.1831, 0.1831, 0)	11	33.3042	360.1 s
ε = 1.5	(0.6317, 0.18415, 0.18415, 0)	11	32.8629	259.8 s
ε = 2	(0.6297, 0.18515, 0.18515, 0)	11	32.4216	2669.1 s
ε = 2.5	(0.6277, 0.1862, 0.1862, 0)	11	31.9802	562.7 s
ε = 5	(0.6172, 0.1914, 0.1914, 0)	9	30.3536	675.8 s
ε = 7.5	(0.6043, 0.19785, 0.19785, 0)	8	28.4175	2347.2 s
ε = 10	(0.5971, 0.20145, 0.20145, 0)	7	26.7566	8157.8 s

## Supplementary Materials

The following are available online at http://www.mdpi.com/1660-4601/15/4/609/s1, Table S1: Related parameters used in practical case study for solving the extended CPEP game, Table S2: Prior probabilities of different chemical plants, Table S3: Attacker results of extended CPEP game under a learning curve of the piecewise function, Table S4: Attacker results of extended CPEP game under a learning curve of the exponential fall function, Table S5: Attacker results of extended CPEP game under a learning curve of the power law function, Table S6: Attacker results computed by COBRA under different rationality levels of attackers.
