# Supplementary material for: An Extended Chemical Plant Environmental Protection Game on Addressing Uncertainties of Human Adversaries
Source: Int J Environ Res Public Health. 2018 Mar 27;15(4):609. doi: 10.3390/ijerph15040609 (PMC5923651; doi:10.3390/ijerph15040609)
Supplement: Supplementary file 1 [file ijerph-15-00609-s001.pdf]

International Journal of
Environmental Research and Public Health

MDPI

Supplementary Materials

Table S1. Related parameters used in practical case study for solving the extended CPEP game.

Chemical Plant	Penalty for Defender	Reward for Attacker
chemical plant a	−368	854
chemical plant b	−361	887
chemical plant c	−353	826
chemical plant d	−351	832
chemical plant e	−391	812
chemical plant f	−393	894
chemical plant g	−365	865
chemical plant h	−396	848
chemical plant i	−374	864
chemical plant j	−373	855
chemical plant k	−357	865
chemical plant l	−376	854
chemical plant m	−380	872
chemical plant n	−366	852
chemical plant o	−363	900
chemical plant p	−374	822
chemical plant q	−383	810
chemical plant r	−393	811
chemical plant s	−371	806
chemical plant t	−387	840
chemical plant u	−398	845
chemical plant v	−362	836
chemical plant w	−388	877
deliberate employee	−400	900

Table S2. Prior probabilities of different chemical plants.

Chemical Plant	Prior Probability	Infraction Number
chemical plant a	0.0303	106
chemical plant b	0.0645	136
chemical plant c	0.0323	68
chemical plant d	0.0517	109
chemical plant e	0.0342	72
chemical plant f	0.0517	109
chemical plant g	0.0527	111
chemical plant h	0.0243	51
chemical plant i	0.0327	69
chemical plant j	0.0313	66
chemical plant k	0.0517	109
chemical plant l	0.0598	126
chemical plant m	0.0371	78
chemical plant n	0.0565	119
chemical plant o	0.0517	109
chemical plant p	0.0214	45
chemical plant q	0.0484	102
chemical plant r	0.0389	82
chemical plant s	0.0626	132
chemical plant t	0.0214	45
chemical plant u	0.0422	89
chemical plant v	0.0404	85
chemical plant w	0.0422	89
deliberate employee	0.02	42

Table S3. Attacker results of extended CPEP game under a learning curve of the piecewise function.

Learning Curve: Piecewise Function											
m = 1		*m* = 2		*m* = 3		*m* = 4		*m* = 5		*m* = 6	
**Att_Str**	**Att_P**	**Att_Str**	**Att_P**	**Att_Str**	**Att_P**	**Att_Str**	**Att_P**	**Att_Str**	**Att_P**	**Att_Str**	**Att_P**
$q_1=1$	235.6	$q_1=1$	202.152	$q_1=1$	109.808	$q_4=1$	−92	$q_4=1$	−96	$q_4=1$	−100
$q_1=1$	281.8	$q_1=1$	247.956	$q_1=1$	154.424	$q_4=1$	−92	$q_4=1$	−96	$q_4=1$	−100
$q_1=1$	196.4	$q_1=1$	163.288	$q_1=1$	71.952	$q_4=1$	−92	$q_4=1$	−96	$q_4=1$	−100
$q_1=1$	204.8	$q_1=1$	171.616	$q_1=1$	80.064	$q_4=1$	−92	$q_4=1$	−96	$q_4=1$	−100
$q_1=1$	176.8	$q_1=1$	143.856	$q_1=1$	53.024	$q_4=1$	−92	$q_4=1$	−96	$q_4=1$	−100
$q_1=1$	291.6	$q_1=1$	257.672	$q_1=1$	163.888	$q_4=1$	−92	$q_4=1$	−96	$q_4=1$	−100
$q_1=1$	251	$q_1=1$	217.42	$q_1=1$	124.68	$q_4=1$	−92	$q_4=1$	−96	$q_4=1$	−100
$q_1=1$	227.2	$q_1=1$	193.824	$q_1=1$	101.696	$q_4=1$	−92	$q_4=1$	−96	$q_4=1$	−100
$q_1=1$	249.6	$q_1=1$	216.032	$q_1=1$	123.328	$q_4=1$	−92	$q_4=1$	−96	$q_4=1$	−100
$q_1=1$	237	$q_1=1$	203.54	$q_1=1$	111.16	$q_4=1$	−92	$q_4=1$	−96	$q_4=1$	−100
$q_1=1$	251	$q_1=1$	217.42	$q_1=1$	124.68	$q_4=1$	−92	$q_4=1$	−96	$q_4=1$	−100
$q_1=1$	235.6	$q_1=1$	202.152	$q_1=1$	109.808	$q_4=1$	−92	$q_4=1$	−96	$q_4=1$	−100
$q_1=1$	260.8	$q_1=1$	227.136	$q_1=1$	134.144	$q_4=1$	−92	$q_4=1$	−96	$q_4=1$	−100
$q_1=1$	232.8	$q_1=1$	199.376	$q_1=1$	107.104	$q_4=1$	−92	$q_4=1$	−96	$q_4=1$	−100
$q_1=1$	300	$q_1=1$	266	$q_1=1$	172	$q_4=1$	−92	$q_4=1$	−96	$q_4=1$	−100
$q_1=1$	190.8	$q_1=1$	157.736	$q_1=1$	66.544	$q_4=1$	−92	$q_4=1$	−96	$q_4=1$	−100
$q_1=1$	174	$q_1=1$	141.08	$q_1=1$	50.32	$q_4=1$	−92	$q_4=1$	−96	$q_4=1$	−100
$q_1=1$	175.4	$q_1=1$	142.468	$q_1=1$	51.672	$q_4=1$	−92	$q_4=1$	−96	$q_4=1$	−100
$q_1=1$	168.4	$q_1=1$	135.528	$q_1=1$	44.912	$q_4=1$	−92	$q_4=1$	−96	$q_4=1$	−100
$q_1=1$	216	$q_1=1$	182.72	$q_1=1$	90.88	$q_4=1$	−92	$q_4=1$	−96	$q_4=1$	−100
$q_1=1$	223	$q_1=1$	189.66	$q_1=1$	97.64	$q_4=1$	−92	$q_4=1$	−96	$q_4=1$	−100
$q_1=1$	210.4	$q_1=1$	177.168	$q_1=1$	85.472	$q_4=1$	−92	$q_4=1$	−96	$q_4=1$	−100
$q_1=1$	267.8	$q_1=1$	234.076	$q_1=1$	140.904	$q_4=1$	−92	$q_4=1$	−96	$q_4=1$	−100
$q_1=1$	300	$q_1=1$	266	$q_1=1$	172	$q_4=1$	−92	$q_4=1$	−96	$q_4=1$	−100

Table S4. Attacker results of extended CPEP game under a learning curve of the exponential fall function.

Learning Curve: Exponential Fall Function											
m = 1		*m* = 2		*m* = 3		*m* = 4		*m* = 5		*m* = 6	
**Att_Str**	**Att_P**	**Att_Str**	**Att_P**	**Att_Str**	**Att_P**	**Att_Str**	**Att_P**	**Att_Str**	**Att_P**	**Att_Str**	**Att_P**
$q_1=1$	235.6	$q_4=1$	−84	$q_4=1$	−88	$q_4=1$	−92	$q_4=1$	−96	$q_4=1$	−100
$q_1=1$	281.8	$q_4=1$	−84	$q_4=1$	−88	$q_4=1$	−92	$q_4=1$	−96	$q_4=1$	−100
$q_1=1$	196.4	$q_4=1$	−84	$q_4=1$	−88	$q_4=1$	−92	$q_4=1$	−96	$q_4=1$	−100
$q_1=1$	204.8	$q_4=1$	−84	$q_4=1$	−88	$q_4=1$	−92	$q_4=1$	−96	$q_4=1$	−100
$q_1=1$	176.8	$q_4=1$	−84	$q_4=1$	−88	$q_4=1$	−92	$q_4=1$	−96	$q_4=1$	−100
$q_1=1$	291.6	$q_4=1$	−84	$q_4=1$	−88	$q_4=1$	−92	$q_4=1$	−96	$q_4=1$	−100
$q_1=1$	251	$q_4=1$	−84	$q_4=1$	−88	$q_4=1$	−92	$q_4=1$	−96	$q_4=1$	−100
$q_1=1$	227.2	$q_4=1$	−84	$q_4=1$	−88	$q_4=1$	−92	$q_4=1$	−96	$q_4=1$	−100
$q_1=1$	249.6	$q_4=1$	−84	$q_4=1$	−88	$q_4=1$	−92	$q_4=1$	−96	$q_4=1$	−100
$q_1=1$	237	$q_4=1$	−84	$q_4=1$	−88	$q_4=1$	−92	$q_4=1$	−96	$q_4=1$	−100
$q_1=1$	251	$q_4=1$	−84	$q_4=1$	−88	$q_4=1$	−92	$q_4=1$	−96	$q_4=1$	−100
$q_1=1$	235.6	$q_4=1$	−84	$q_4=1$	−88	$q_4=1$	−92	$q_4=1$	−96	$q_4=1$	−100
$q_1=1$	260.8	$q_4=1$	−84	$q_4=1$	−88	$q_4=1$	−92	$q_4=1$	−96	$q_4=1$	−100

$q_1=1$	232.8	$q_4=1$	−84	$q_4=1$	−88	$q_4=1$	−92	$q_4=1$	−96	$q_4=1$	−100
$q_1=1$	300	$q_1=1$	−79	$q_4=1$	−88	$q_4=1$	−92	$q_4=1$	−96	$q_4=1$	−100
$q_1=1$	190.8	$q_4=1$	−84	$q_4=1$	−88	$q_4=1$	−92	$q_4=1$	−96	$q_4=1$	−100
$q_1=1$	174	$q_4=1$	−84	$q_4=1$	−88	$q_4=1$	−92	$q_4=1$	−96	$q_4=1$	−100
$q_1=1$	175.4	$q_4=1$	−84	$q_4=1$	−88	$q_4=1$	−92	$q_4=1$	−96	$q_4=1$	−100
$q_1=1$	168.4	$q_4=1$	−84	$q_4=1$	−88	$q_4=1$	−92	$q_4=1$	−96	$q_4=1$	−100
$q_1=1$	216	$q_4=1$	−84	$q_4=1$	−88	$q_4=1$	−92	$q_4=1$	−96	$q_4=1$	−100
$q_1=1$	223	$q_4=1$	−84	$q_4=1$	−88	$q_4=1$	−92	$q_4=1$	−96	$q_4=1$	−100
$q_1=1$	210.4	$q_4=1$	−84	$q_4=1$	−88	$q_4=1$	−92	$q_4=1$	−96	$q_4=1$	−100
$q_1=1$	267.8	$q_4=1$	−84	$q_4=1$	−88	$q_4=1$	−92	$q_4=1$	−96	$q_4=1$	−100
$q_1=1$	300	$q_1=1$	−79	$q_4=1$	−88	$q_4=1$	−92	$q_4=1$	−96	$q_4=1$	−100

Table S5. Attacker results of extended CPEP game under a learning curve of the power law function.

Learning curve: exponential fall function											
m = 1		*m* = 2		*m* = 3		*m* = 4		*m* = 5		*m* = 6	
**Att_Str**	**Att_P**	**Att_Str**	**Att_P**	**Att_Str**	**Att_P**	**Att_Str**	**Att_P**	**Att_Str**	**Att_P**	**Att_Str**	**Att_P**
$q_1=1$	235.6	$q_4=1$	−84	$q_4=1$	−88	$q_4=1$	−92	$q_4=1$	−96	$q_4=1$	−100
$q_1=1$	281.8	$q_1=1$	−79	$q_4=1$	−88	$q_4=1$	−92	$q_4=1$	−96	$q_4=1$	−100
$q_1=1$	196.4	$q_4=1$	−84	$q_4=1$	−88	$q_4=1$	−92	$q_4=1$	−96	$q_4=1$	−100
$q_1=1$	204.8	$q_4=1$	−84	$q_4=1$	−88	$q_4=1$	−92	$q_4=1$	−96	$q_4=1$	−100
$q_1=1$	176.8	$q_4=1$	−84	$q_4=1$	−88	$q_4=1$	−92	$q_4=1$	−96	$q_4=1$	−100
$q_1=1$	291.6	$q_1=1$	−70.204	$q_4=1$	−88	$q_4=1$	−92	$q_4=1$	−96	$q_4=1$	−100
$q_1=1$	251	$q_4=1$	−84	$q_4=1$	−88	$q_4=1$	−92	$q_4=1$	−96	$q_4=1$	−100
$q_1=1$	227.2	$q_4=1$	−84	$q_4=1$	−88	$q_4=1$	−92	$q_4=1$	−96	$q_4=1$	−100
$q_1=1$	249.6	$q_4=1$	−84	$q_4=1$	−88	$q_4=1$	−92	$q_4=1$	−96	$q_4=1$	−100
$q_1=1$	237	$q_4=1$	−84	$q_4=1$	−88	$q_4=1$	−92	$q_4=1$	−96	$q_4=1$	−100
$q_1=1$	251	$q_4=1$	−84	$q_4=1$	−88	$q_4=1$	−92	$q_4=1$	−96	$q_4=1$	−100
$q_1=1$	235.6	$q_4=1$	−84	$q_4=1$	−88	$q_4=1$	−92	$q_4=1$	−96	$q_4=1$	−100
$q_1=1$	260.8	$q_4=1$	−84	$q_4=1$	−88	$q_4=1$	−92	$q_4=1$	−96	$q_4=1$	−100
$q_1=1$	232.8	$q_4=1$	−84	$q_4=1$	−88	$q_4=1$	−92	$q_4=1$	−96	$q_4=1$	−100
$q_1=1$	300	$q_1=1$	−62.665	$q_4=1$	−88	$q_4=1$	−92	$q_4=1$	−96	$q_4=1$	−100
$q_1=1$	190.8	$q_4=1$	−84	$q_4=1$	−88	$q_4=1$	−92	$q_4=1$	−96	$q_4=1$	−100
$q_1=1$	174	$q_4=1$	−84	$q_4=1$	−88	$q_4=1$	−92	$q_4=1$	−96	$q_4=1$	−100
$q_1=1$	175.4	$q_4=1$	−84	$q_4=1$	−88	$q_4=1$	−92	$q_4=1$	−96	$q_4=1$	−100
$q_1=1$	168.4	$q_4=1$	−84	$q_4=1$	−88	$q_4=1$	−92	$q_4=1$	−96	$q_4=1$	−100
$q_1=1$	216	$q_4=1$	−84	$q_4=1$	−88	$q_4=1$	−92	$q_4=1$	−96	$q_4=1$	−100
$q_1=1$	223	$q_4=1$	−84	$q_4=1$	−88	$q_4=1$	−92	$q_4=1$	−96	$q_4=1$	−100
$q_1=1$	210.4	$q_4=1$	−84	$q_4=1$	−88	$q_4=1$	−92	$q_4=1$	−96	$q_4=1$	−100
$q_1=1$	267.8	$q_4=1$	−84	$q_4=1$	−88	$q_4=1$	−92	$q_4=1$	−96	$q_4=1$	−100
$q_1=1$	300	$q_1=1$	−62.665	$q_4=1$	−88	$q_4=1$	−92	$q_4=1$	−96	$q_4=1$	−100

Table S6. Attacker results computed by COBRA under different rationality levels of attackers.

ε = 0.5		ε = 1		ε = 1.5		ε = 2		ε = 2.5		ε = 5		ε = 7.5		ε = 10	
**Att_Str**	**Att_P**	**Att_Str**	**Att_P**	**Att_Str**	**Att_P**	**Att_Str**	**Att_P**	**Att_Str**	**Att_P**	**Att_Str**	**Att_P**	**Att_Str**	**Att_P**	**Att_Str**	**Att_P**
$q_1 = 1$	−84.45	$q_1 = 1$	−83.45	$q_1 = 1$	−82.45	$q_1 = 1$	−81.45	$q_1 = 1$	−80.45	$q_1 = 1$	−75.34	$q_1 = 1$	−68.99	$q_1 = 1$	−65.44
$q_1 = 1$	−42.45	$q_1 = 1$	−41.44	$q_1 = 1$	−40.42	$q_1 = 1$	−39.41	$q_1 = 1$	−38.39	$q_1 = 1$	−33.21	$q_1 = 1$	−26.78	$q_1 = 1$	−23.18
$q_4 = 1$	−88	$q_4 = 1$	−88.00	$q_4 = 1$	−88.00	$q_4 = 1$	−88.00	$q_4 = 1$	−88.00	$q_4 = 1$	−88.00	$q_4 = 1$	−88.00	$q_4 = 1$	−88.00
$q_4 = 1$	−88	$q_4 = 1$	−88.00	$q_4 = 1$	−88.00	$q_4 = 1$	−88.00	$q_4 = 1$	−88.00	$q_4 = 1$	−88.00	$q_4 = 1$	−88.00	$q_4 = 1$	−88.00
$q_4 = 1$	−88	$q_4 = 1$	−88.00	$q_4 = 1$	−88.00	$q_4 = 1$	−88.00	$q_4 = 1$	−88.00	$q_4 = 1$	−88.00	$q_4 = 1$	−88.00	$q_4 = 1$	−88.00
$q_1 = 1$	−33.54	$q_1 = 1$	−32.52	$q_1 = 1$	−31.51	$q_1 = 1$	−30.49	$q_1 = 1$	−29.47	$q_1 = 1$	−24.28	$q_1 = 1$	−17.82	$q_1 = 1$	−14.22
$q_1 = 1$	−70.45	$q_1 = 1$	−69.45	$q_1 = 1$	−68.44	$q_1 = 1$	−67.44	$q_1 = 1$	−66.43	$q_1 = 1$	−61.30	$q_1 = 1$	−54.92	$q_1 = 1$	−51.35
$q_4 = 1$	−88	$q_4 = 1$	−88.00	$q_4 = 1$	−88.00	$q_4 = 1$	−88.00	$q_4 = 1$	−88.00	$q_1 = 1$	−83.00	$q_1 = 1$	−76.66	$q_1 = 1$	−73.12
$q_1 = 1$	−71.73	$q_1 = 1$	−70.72	$q_1 = 1$	−69.72	$q_1 = 1$	−68.71	$q_1 = 1$	−67.71	$q_1 = 1$	−62.58	$q_1 = 1$	−56.20	$q_1 = 1$	−52.63
$q_1 = 1$	−83.18	$q_1 = 1$	−82.18	$q_1 = 1$	−81.18	$q_1 = 1$	−80.18	$q_1 = 1$	−79.18	$q_1 = 1$	−74.06	$q_1 = 1$	−67.71	$q_1 = 1$	−64.16
$q_1 = 1$	−70.45	$q_1 = 1$	−69.45	$q_1 = 1$	−68.44	$q_1 = 1$	−67.44	$q_1 = 1$	−66.43	$q_1 = 1$	−61.30	$q_1 = 1$	−54.92	$q_1 = 1$	−51.35
$q_1 = 1$	−84.45	$q_1 = 1$	−83.45	$q_1 = 1$	−82.45	$q_1 = 1$	−81.45	$q_1 = 1$	−80.45	$q_1 = 1$	−75.34	$q_1 = 1$	−68.99	$q_1 = 1$	−65.44
$q_1 = 1$	−61.54	$q_1 = 1$	−60.54	$q_1 = 1$	−59.53	$q_1 = 1$	−58.52	$q_1 = 1$	−57.51	$q_1 = 1$	−52.36	$q_1 = 1$	−45.96	$q_1 = 1$	−42.39
$q_1 = 1$	−87	$q_1 = 1$	−86.00	$q_1 = 1$	−85.00	$q_1 = 1$	−84.00	$q_1 = 1$	−83.00	$q_1 = 1$	−77.89	$q_1 = 1$	−71.55	$q_1 = 1$	−68.00
$q_1 = 1$	−25.90	$q_1 = 1$	−24.88	$q_1 = 1$	−23.86	$q_1 = 1$	−22.85	$q_1 = 1$	−21.83	$q_1 = 1$	−16.62	$q_1 = 1$	−10.15	$q_1 = 1$	−6.53
$q_4 = 1$	−88	$q_4 = 1$	−88.00	$q_4 = 1$	−88.00	$q_4 = 1$	−88.00	$q_4 = 1$	−88.00	$q_4 = 1$	−88.00	$q_4 = 1$	−88.00	$q_1 = 1$	−87.21
$q_4 = 1$	−88	$q_4 = 1$	−88.00	$q_4 = 1$	−88.00	$q_4 = 1$	−88.00	$q_4 = 1$	−88.00	$q_4 = 1$	−88.00	$q_4 = 1$	−88.00	$q_4 = 1$	−88.00
$q_4 = 1$	−88	$q_4 = 1$	−88.00	$q_4 = 1$	−88.00	$q_4 = 1$	−88.00	$q_4 = 1$	−88.00	$q_4 = 1$	−88.00	$q_4 = 1$	−88.00	$q_4 = 1$	−88.00
$q_4 = 1$	−88	$q_4 = 1$	−88.00	$q_4 = 1$	−88.00	$q_4 = 1$	−88.00	$q_4 = 1$	−88.00	$q_4 = 1$	−88.00	$q_4 = 1$	−88.00	$q_4 = 1$	−88.00
$q_4 = 1$	−88	$q_4 = 1$	−88.00	$q_4 = 1$	−88.00	$q_4 = 1$	−88.00	$q_4 = 1$	−88.00	$q_4 = 1$	−88.00	$q_1 = 1$	−86.90	$q_1 = 1$	−83.37
$q_4 = 1$	−88	$q_4 = 1$	−88.00	$q_4 = 1$	−88.00	$q_4 = 1$	−88.00	$q_4 = 1$	−88.00	$q_1 = 1$	−86.83	$q_1 = 1$	−80.50	$q_1 = 1$	−76.96
$q_4 = 1$	−88	$q_4 = 1$	−88.00	$q_4 = 1$	−88.00	$q_4 = 1$	−88.00	$q_4 = 1$	−88.00	$q_4 = 1$	−88.00	$q_4 = 1$	−88.00	$q_4 = 1$	−88.00
$q_1 = 1$	−55.18	$q_1 = 1$	−54.17	$q_1 = 1$	−53.16	$q_1 = 1$	−52.15	$q_1 = 1$	−51.14	$q_1 = 1$	−45.98	$q_1 = 1$	−39.57	$q_1 = 1$	−35.99
$q_1 = 1$	−25.90	$q_1 = 1$	−24.88	$q_1 = 1$	−23.86	$q_1 = 1$	−22.85	$q_1 = 1$	−21.83	$q_1 = 1$	−16.62	$q_1 = 1$	−10.15	$q_1 = 1$	−6.53
